# N^6^-methyladenosine METTL3 promotes cervical cancer tumorigenesis and Warburg effect through YTHDF1/HK2 modification

**DOI:** 10.1038/s41419-020-03071-y

**Published:** 2020-10-24

**Authors:** Qianqing Wang, Xiangcui Guo, Li Li, Zhihui Gao, Xiaoke Su, Mei Ji, Juan Liu

**Affiliations:** 1 Department of Gynecology, Xinxiang Central Hospital, 453000 Xinxiang, Henan China; 2 Department of Gynecology, The First Affiliated Hospital of Zhengzhou University, 450003 Zhengzhou, China; 3 Department of Gynecology, Third Affiliated Hospital Guangzhou Medical University, 510080 Guangdong, China

**Keywords:** Glycobiology, Cancer metabolism

## Abstract

N6-methyladenosine (m^6^A) serves as the most common and conserved internal transcriptional modification. However, the roles of m^6^A on cervical cancer (CC) tumorigenesis are still unclear. Here, results indicated that METTL3 was significantly upregulated in CC tissue and cells, which was closely correlated with the lymph node metastasis and poor prognosis of CC patients. MeRIP-Seq analysis revealed the m^6^A profiles in CC cells. Functionally, METTL3 promoted the proliferation and Warburg effect (aerobic glycolysis) of CC cells. Mechanistically, METTL3 targeted the 3’-Untranslated Region (3’-UTR) of hexokinase 2 (HK2) mRNA. Moreover, METTL3 recruited YTHDF1, a m^6^A reader, to enhance HK2 stability. These findings demonstrated that METTL3 enhanced the HK2 stability through YTHDF1-mediated m^6^A modification, thereby promoting the Warburg effect of CC, which might promote a novel insight for the CC treatment.

## Introduction

Cervical cancer (CC) acts as one of the commonest malignancies in human cancer, emerging as an enormous challenge for women worldwide^1,2^. However, there are large amount of by reason of uneffective methods for early diagnosis^3^. Accumulating evidences have revealed that CC tumorigenesis is a multistep process involving complicated interplays of transcriptomic alterations, genetics, and epigenetics^4^. Although more and more efforts are invested for the CC treatments, the clinical outcomes for CC patients are still dismal in current treatment strategies^5^. The main therapies for advanced CC are palliative surgery incorporated by systemic chemotherapy. In spite of this, the overall postoperative survival rate is still short-lived. Therefore, the investigation for CC pathogenesis molecular mechanisms is essential for its development.

N^6^-methyladenosine (m^6^A) is the most prevalent modification in mammalian mRNA, as well as in rRNA and tRNA^6,7^. m^6^A is a reversible and dynamic modification for mRNA, which could control of mRNA metabolism^8–10^. The installation of m^6^A is mediated by m^6^A methyltransferase, including methyltransferase-like 3 (METTL3), methyltransferase-like 14 (METTL14), Wilms-tumour associated protein (WTAP)^11^. The uninstallation of m^6^A is operated by demethylase, including FTO and ALKBH5. Besides, m^6^A reader protein, YTH domain family, could recognize the m^6^A-modified mRNA^12^. The m^6^A motif sequences are DRACH (D = A/G/U, R = A/G, H = A/C/U). The modification of m^6^A could regulate the tumorigenesis of human cancer. For example, in colorectal carcinoma, METTL3 is highly expressed in metastatic tissues and prevent SOX2 mRNA degradation by specifically cooperate with m^6^A reader IGF2BP2^13^.

Warburg effect, also known as aerobic glycolysis, is a typical hallmark for cancer metabolism^14–16^. Although the cellular oxygen is adequate, tumor cells still insist on the aerobic glycolysis to generate energy rather than the mitochondrial oxidative phosphorylation^17^. The characteristic energy supplement is identified as Warburg effect. In CC tumorigenesis, the means to repress the Warburg effect are considered as effective therapeutic approach^18^. In this research, we focused on the roles of METTL3 in the CC tumorigenesis and Warburg effect. Results demonstrated that the high expression of METTL3 indicated the poor prognosis. METTL3 promoted the proliferation and Warburg effect of CC cells by enhancing HK2 stability through recruiting YTHDF1. These findings might promote a novel insight for the CC treatment.

## Materials and methods

### Patients’ tissue samples collection

The study protocol was approved by the Xinxiang Central Hospital of Ethics Committee and conducted in accordance with the declaration of Helsinki and German Federal Guidelines. CC tissue samples were collected during the surgery (Table 1).

**Table 1:** Relationship between METTL3 and CC patients’ clinicopathological characteristic.

		60	METTL3		*p* value
			Low = 29	High = 31	
Age	<50	37	18	19	0.525
	≥50	23	11	12	
Lymph node metastasis	Yes	35	12	23	0.001*
	No	25	17	8	
Histological type	Malignant	33	8	25	0.001*
	Normal	27	21	6	
Tumor size	≥3 cm	41	21	20	0.318
	<3 cm	19	8	11	
FIGO stage	I–II	24	15	9	0.005*
	II–IV	36	14	22	

*P < 0.05 represents statistical differences.

### Cell lines and culture

The cervical cancer cell lines (CaSki, SiHa, C33A, HT-3) as well as epidermal cell (HaCaT) were provided by Cell Bank of Type Culture Collection of Chinese Academy of Sciences (Shanghai, China) and maintained in RPMI 1640 medium (Wisent, Shanghai, China) supplemented with 10% fetal bovine serum (FBS, Gibco, Gran Island, NY, USA), 100 U/ml of penicillin, and 100 μg/ml of streptomycin (HyClone) in a humidified atmosphere containing 5% CO_2_ at 37 °C.

### Cellular transfection

The short hairpin RNA (shRNA) for METTL3 and negative control were designed to generate the hairpin RNA (shRNA) by Shanghai Genechem Co., Ltd., (Shanghai, China). SiHa and CaSki cells were seeded into 6-well plates and the transfections for pLKO.1 plasmids in CC cells were performed using the Lipofectamine 2000 kit (Invitrogen, Carlsbad, CA, USA) following the manufacturer’s instructions. The sequences were listed in Table S1.

### Quantitative real-time PCR (qRT-PCR)

Total RNAs were extracted from CC tissue and cells by using TRIzol. The purity and concentration of the RNA were examined spectrophotometrically by measuring the absorbance at 260/280 nm (ratio 1.8–2.1) using the NanoDrop ND-1000 (Thermo Fisher Scientific, Wilmington, DE, USA). Reverse transcription was conducted for cDNA amplification using RNA (1 μg) using SuperScript II Reverse Transcriptase (Invitrogen, Thermo Fisher Scientific). PCR was performed using the SYBR Green Master Mix kit (Takara, Otsu, Japan) on AB7300 thermo-recycler (Applied Biosystems, Carlsbad, CA, USA) with primers. For quantitative RT-PCR, GAPDH was used as endogenous control using the 2^−ΔΔCT^ method. The primers used are listed in supplementary Table S1.

### Western blot analysis

Protein extraction was performed using protein extraction kit (Key Gene, KGP9100) form CC cells. Lipid proteins were appended into gels and subjected nitrocellulose membranes. Total proteins were harvested as indicated in figure legends, separated using sodium dodecyl sulfate polyacrylamide gel electrophoresis (SDS-PAGE). The nitrocellulose membrane was incubated with antibodies against METTL3 (ab195352, Abcam, 1:1000), against YTHDF1 (ab220162), against HK2 (ab209847, Abcam, 1:1000). GAPDH was used as normalized control.

### Glucose uptake, lactate production, and ATP levels

The glucose uptake level was quantified using glucose assay kit (Sigma, St-Louis, MO, USA). The lactate level was quantified using the Lactate Assay kit (BioVision, Mountain View, CA, USA). The ATP level was quantified using CellTiter-Glo Luminescent Cell Viability Assay (Promega, Madison, MI, USA).

### Extracellular acidification rate assay

The extracellular acidification rate (ECAR) was determined using the Seahorse XF 96 Extracellular Flux Analyzer (Agilent Technologies, Santa Clara, CA, USA) according to the manufacturer’s instructions as previously described^19^.

### Proliferative assay

Proliferative assay for CC cells was detected using CCK-8 assay and colony formation assay. For CCK-8 assay, cells were seeded in 96-well plates at a density of 1 × 10^3^ cells per well and then administrated with CCK-8 Kit (Dojindo, Kumamoto, Japan) at indicated time points (1, 2, 3, and 4 days). The absorbance at 450 nm was measured. For colony formation assay, about 1000 transfected cells were seeded into 6-well plates and then cultured for 14 days. Then, cells were fixed with methanol and stained with 0.1% crystal violet. Three duplicates were performed.

### Flow cytometry analysis

Apoptosis and cycle analysis of CC cells were analyzed using flow cytometry analysis. For the apoptosis, SiHa and CaSki were harvested in binding buffer (200 ml) and then stained with AnnexinV-FITC (5 μl) and propidium iodide (PI, 5 μl) for 15 min using an apoptosis Kit (KeyGen, China). After incubation with Binding Buffer (400 μl), the cells were subjected to flow cytometric analysis using FACS Canto II flow cytometry (BD Biosciences). For the cycle analysis, cells were fixed in 75% ethanol overnight at 4 °C and stained with PI in PBS according to the manufacturer’s instructions. The stained cells were analyzed by flow cytometry for cycle analysis.

### m^6^A quantitative measurement

The quantification assay for m^6^A was performed as previously described^20^. Total RNAs were isolated by TRIzol from CC cells according to the manufacturer’s instructions. For the m^6^A quantitation, m^6^A RNA methylation quantification kit (ab185912, Abcam) was performed. The m^6^A content level was quantified colorimetrically by reading the absorbance at 450 nm.

### Methylated RNA immune-precipitation qPCR (MeRIP-qPCR)

MeRIP-qPCR assay was performed as previously described^20^. Total RNA was extracted from CC cells and then conjugated with protein A/G magnetic beads in IP buffer, containing anti-m^6^A antibody (ab208577, Abcam) and anti-IgG, supplemented with RNase inhibitor and protease inhibitor overnight at 4 °C. The m^6^A-modified HK2 level was precipitated. The relative enrichment was calculated by calculating the 2^−ΔΔCt^ compared to the input sample.

### RNA stability

The RNA was extracted from CC cells using Trizol. Then, the RNA was treated with actinomycin D (Act D, 1 μg/ml) and then the HK2 mRNA level was measured using qRT-PCR at indicated timepoint.

### Animal experiment

Five-week-old male nude BALB/C mice were applied for this animal studies and fed with certified standard diet and tap water ad libitum in a light/dark cycle of 12 h on/12 h off. This animal assay was approved by the Institutional Animal Care and Use Committee of the Xinxiang Central Hospital. The assay was performed in accordance with the National Institutes of Health Guide for the Care and Use of Laboratory Animals. Stable transfection of METTL3 knockdown (sh-METTL3) or negative control (sh-blank) in SiHa cells (5 × 10^6^ cells per 0.1 mL) were injected into the flank of mice. The tumor size was measured once three days using the formula (0.5 × length × width^2^) and the weight was detected after 3 weeks.

### Statistical analysis

All the data were statistically analyzed using SPSS software (19.0 version, Chicago, IL, USA). Comparison of continuous variables was performed by Student’s *t*-test. The Kaplan–Meier methods and log-rank test were used to calculate the survival curves of CC patients. *P* value less than 0.05 was considered as statistical significance.

## Results

### METTL3 regulates the m^6^A level in CC tissue and cells

**Fig. 1:**
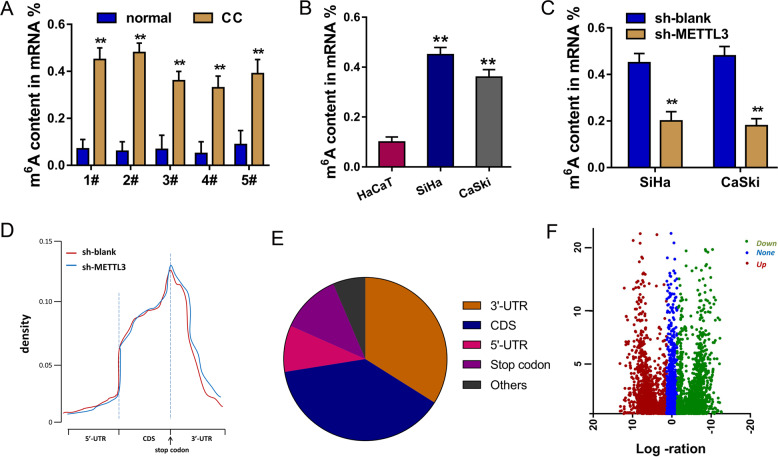
METTL3 regulates the m^6^A level in CC tissue and cells. **a** M^6^A quantitative analysis showed the percentage of m^6^A content in the CC tissue and adjacent normal tissue. **b** M^6^A quantitative analysis showed the percentage of m^6^A content in CC cells (SiHa, CaSki) and normal cells (HaCaT). **c** M^6^A quantitative analysis showed the percentage of m^6^A content in CC cells (SiHa, CaSki) transfected with METTL3 knockdown. **d** MeRIP-Seq analysis revealed the m^6^A peaks distributed at 3’-UTR, 5’-UTR and coding sequence (CDS). **e** Proportion analysis illustrated the m^6^A peaks were distributed in the 3’-UTR, CDS, 5’-UTR and stop codon. **f** Volcano plot of MeRIP-Seq showed the dysregulated m^6^A peaks in the CC cells. Data are displayed as mean ± standard deviation. **p < 0.01 vs. control.

In the CC tissue samples, the level of m^6^A was found to be upregulated as compared to the normal control tissue (Fig. 1a). Analogously, the level of m^6^A in CC cells was increased in the CC cells (Fig. 1b). In the CC cells, METTL3 knockdown transfection could reduce the m^6^A level (Fig. 1c). MeRIP-Seq analysis revealed that m^6^A peaks distributed at 3’-UTR, 5’-UTR and coding sequence (CDS). Particularly, the m^6^A peaks were mainly located in the surrounding area of stop codon (Fig. 1d). Besides, the proportion analysis illustrated that m^6^A peaks were distributed in the 3’-UTR, CDS, 5’-UTR, and stop codon (Fig. 1e). Volcano plot of MeRIP-Seq showed that there were hundreds of m^6^A peaks in the CC cells (Fig. 1f). In conclusion, these findings unveil that METTL3 regulates the m^6^A level in CC tissue and cells.

### METTL3 and YTHDF1 indicate the poor prognosis of CC patients

**Fig. 2:**
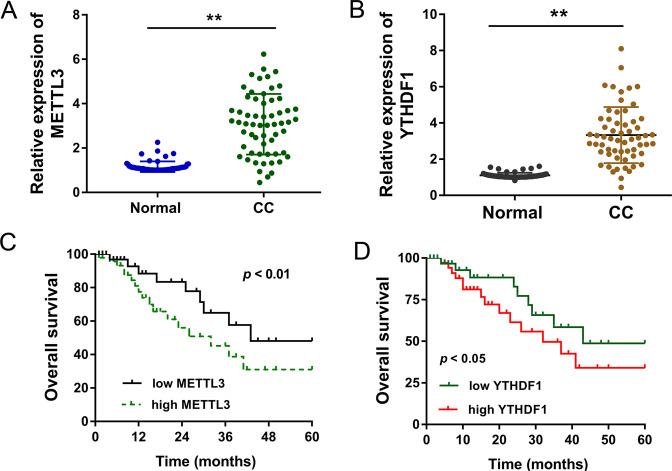
METTL3 and YTHDF1 indicate the poor prognosis of CC patients. **a** RT-PCR indicated the METTL3 mRNA in the CC tissue samples and normal control. **b** RT-PCR indicated the YTHDF1 level in the CC tissue and normal samples. **c** Prognosis analysis indicated the survival rate of CC patients with high or low METTL3 level. **d** Survival of CC patients with high or low YTHDF1 in collected CC cohort. Data are displayed as mean ± standard deviation. **p < 0.01 vs. control. *p < 0.05 vs. control.

In the CC tissue samples, METTL3 mRNA was found to be upregulated as compared to the normal control (Fig. 2a). Moreover, for a critical m^6^A reader protein YTHDF1, its level in CC tissue was significantly upregulated as compared to the normal samples (Fig. 2b). In CC cohort, higher METTL3 indicated the poor survival of CC patients (Fig. 2c). In CC cohort, higher YTHDF1 level indicated the poor survival of CC patients (Fig. 2d). In conclusion, METTL3 and YTHDF1 indicate the poor prognosis of CC patients.

### METTL3 promotes the glycolysis of CC cells

**Fig. 3:**
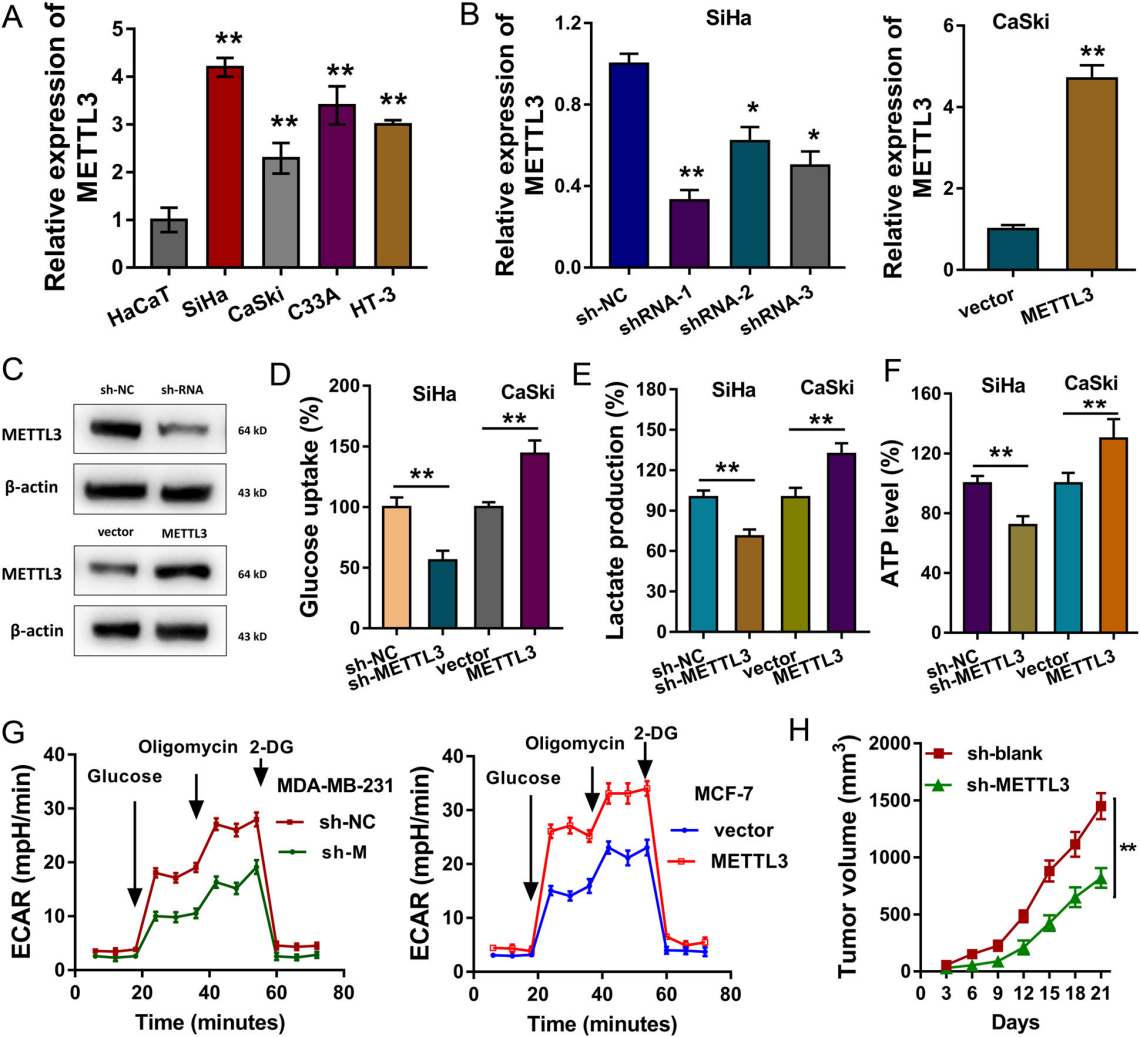
METTL3 promotes the glycolysis of CC cells. **a** RT-PCR revealed the METTL3 mRNA level in CC cell lines (SiHa, CaSki, C33A, HT-3) and normal cell line (HaCaT). **b** The knockdown and overexpression of METTL3 were constructed for the functional experiments. **c** Western blot analysis indicated the knockdown and overexpression efficiency for the transfection. **e** Glucose uptake analysis, **f** lactate production analysis, and **g** ATP level analysis were analyzed. **g** Extracellular acidification rate (ECAR) profiles were detected for the glycolytic capacity of METTL3 knockdown and METTL3 overexpression. **h** Xenograft mice assay in vivo demonstrated the tumor volume of cells transfected with METTL3 knockdown. Data are displayed as mean ± standard deviation. **p < 0.01 vs. control. *p < 0.05 vs. control.

In the CC cell lines, RT-PCR indicated that METTL3 mRNA level was increased as compared to the normal cells (Fig. 3a). The knockdown and overexpression of METTL3 were constructed for the functional experiments (Fig. 3b). Western blot analysis indicated the knockdown and overexpression efficiency for the transfection (Fig. 3c). Energy metabolism analysis indicated that METTL3 overexpression promoted the glucose uptake, lactate production and ATP level, while METTL3 knockdown repressed them (Fig. 3d–f). Extracellular acidification rate (ECAR) assay showed that METTL3 markedly promoted the glycolytic capacity, while METTL3 knockdown decreased it (Fig. 3g). In vivo xenograft mice assay was performed and results demonstrated that METTL3 knockdown could inhibit the tumor growth (Fig. 3h). Overall, these findings reveal that METTL3 promotes the glycolysis of CC cells.

### METTL3 promotes the proliferation of CC cells in vitro

**Fig. 4:**
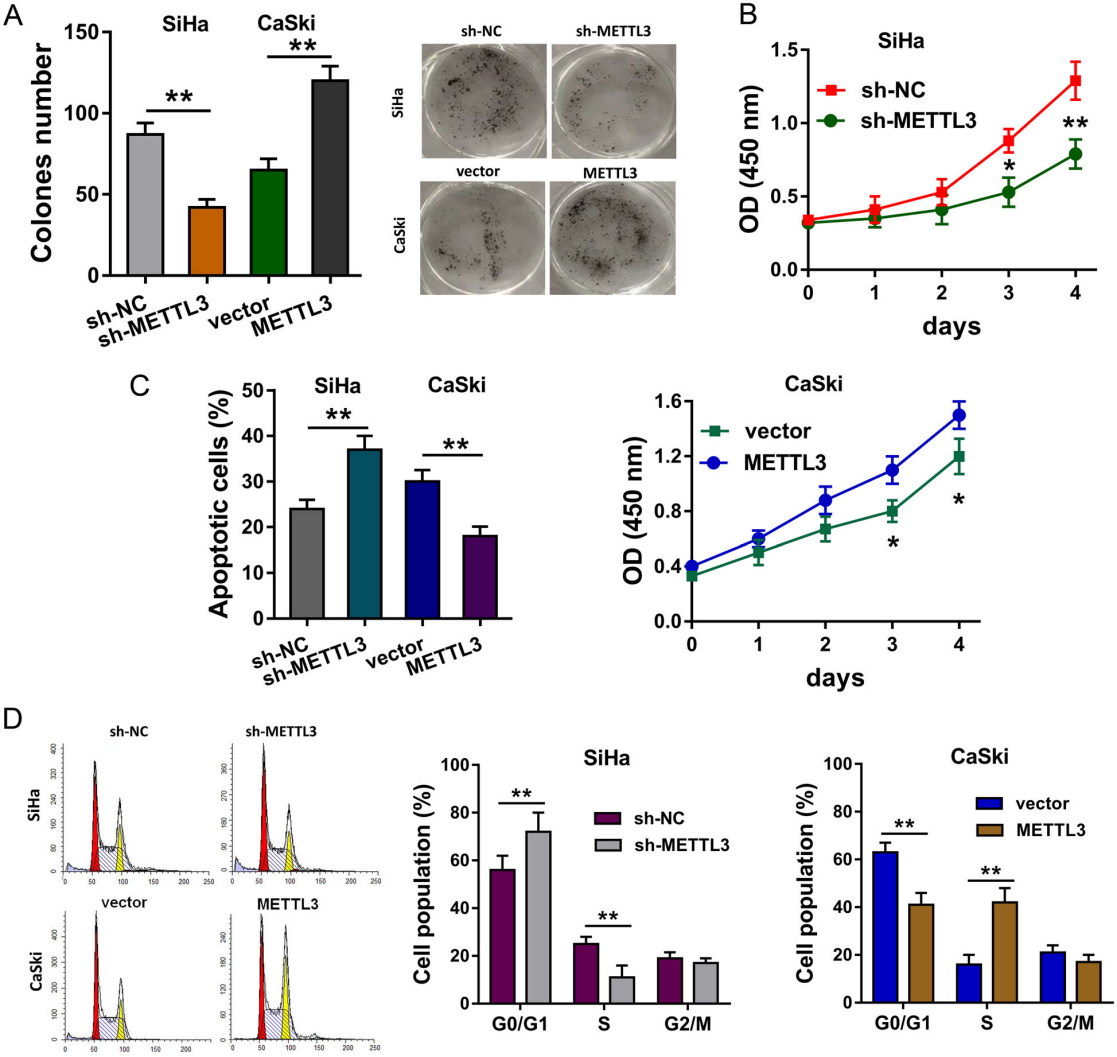
METTL3 promotes the proliferation and invasion of CC cells. **a** Colony formation assay indicated the clones’ number of CC cells. SiHa cells were transfected with METTL3 knockdown, and CaSki cells were transfected with METTL3 overexpression. **b** CCK-8 assay illustrated the proliferative ability with METTL3 knockdown or METTL3 overexpression. **c** Flow cytometry for apoptosis indicated the apoptotic rate with METTL3 knockdown and METTL3 overexpression. **d** Flow cytometry for cell-cycle analysis demonstrated the cellular distribution at G0/G1, S, G2/M phase. Data are displayed as mean ± standard deviation. **p < 0.01 vs. control. *p < 0.05 vs. control.

For the cellular functional experiments, the gain and loss of function assays were performed using the METTL3 silencing in SiHa cell line or overexpression transfection in CaSki cell line. Colony formation assay indicated that METTL3 knockdown repressed the clones’ number of CC cells, while METTL3 overexpression promoted it (Fig. 4a). CCK-8 assay illustrated that METTL3 knockdown inhibited the proliferative ability of SiHa cells, and METTL3 overexpression accelerated it of CaSki cells (Fig. 4b). Flow cytometry for apoptosis indicated that METTL3 knockdown increased the apoptotic rate, while METTL3 overexpression reduced it (Fig. 4c). Flow cytometry for cell-cycle analysis demonstrated that METTL3 knockdown induced the cycle arrest at G0/G1 phase, while METTL3 overexpression promoted the cycle progression (Fig. 4d). In conclusion, these findings support the conclusion that METTL3 promotes the proliferation of CC cells in vitro.

### HK2 acts as the target of METTL3 in CC cells

**Fig. 5:**
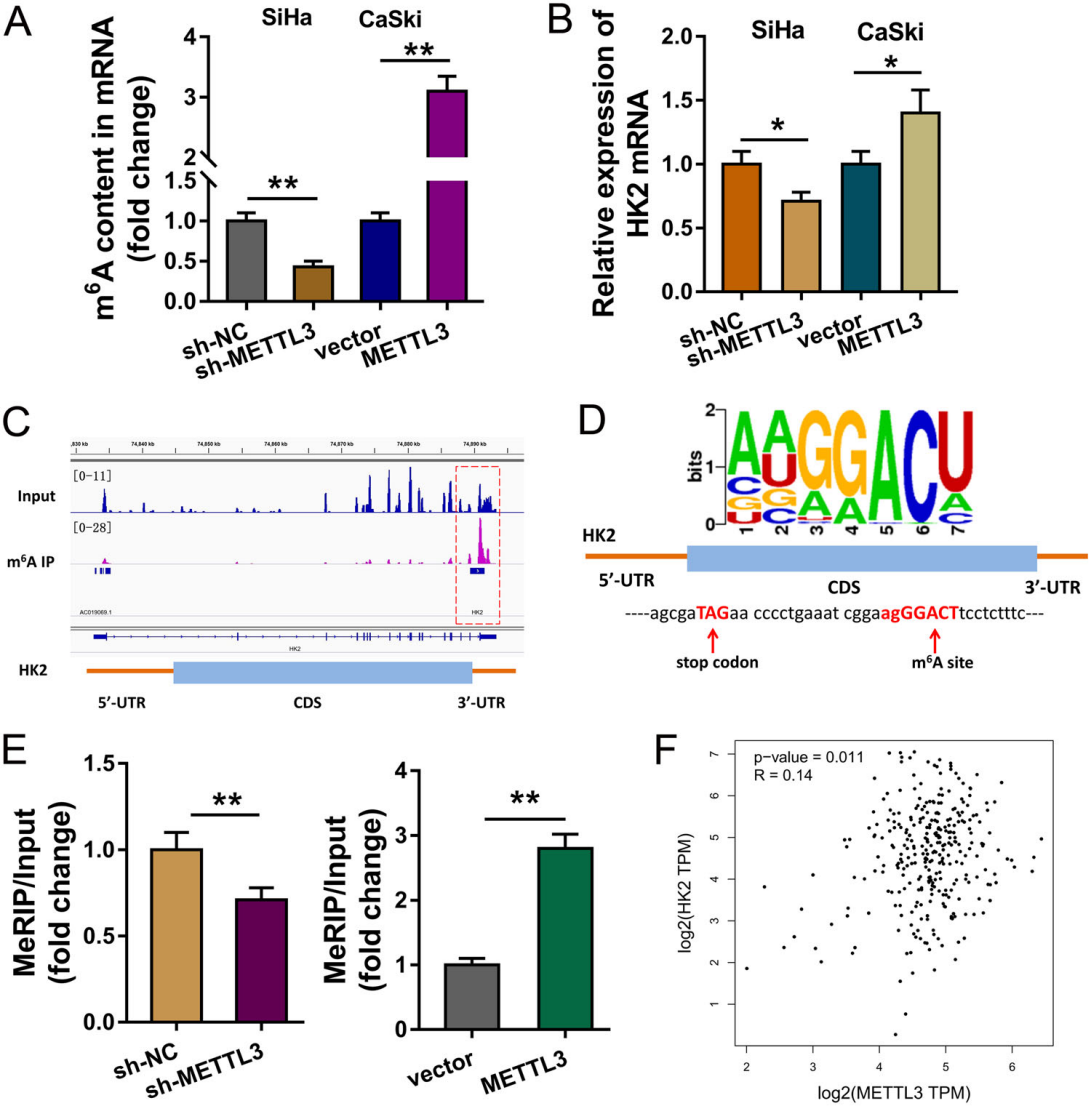
HK2 acts as the target of METTL3 in CC cells. **a** m^6^A content quantitative analysis indicated the m^6^A content level in the transfection of METTL3 knockdown or METTL3 overexpression. **b** RT-PCR indicated the HK2 mRNA in the transfection of METTL3 knockdown or METTL3 overexpression. **c** MeRIP-Seq analysis was performed using SiHa cells and results indicated the m^6^A site in the 3’-UTR of HK2 mRNA. **d** Schematic diagram demonstrated the m^6^A motif of METTL3 and the m^6^A site in the 3’-UTR of HK2 mRNA (near stop codon). **e** MeRIP-qPCR indicated the HK2 mRNA enrichment precipitated by m^6^A antibody. **f** Correlation analysis by Spearman’s rank correlation coefficient (GEPIA, http://gepia.cancer-pku.cn/) showed the correlation within METTL3 and HK2 in the CC tissue specimens. Data are displayed as mean ± standard deviation. **p < 0.01 vs. control. *p < 0.05 vs. control.

To discover the roles of METTL3 on the CC cells, the m^6^A level was detected using the m^6^A content quantitative analysis (Fig. 5a). Results indicated that METTL3 knockdown repressed the m^6^A content level, while METTL3 overexpression increased m^6^A content. RT-PCR indicated that METTL3 knockdown decreased the HK2 mRNA and METTL3 overexpression promoted the HK2 mRNA level (Fig. 5b). MeRIP-Seq analysis was performed using SiHa cells and results indicated that there was a m^6^A site in the 3’-UTR of HK2 mRNA (Fig. 5c). Schematic diagram demonstrated the m^6^A motif of METTL3 and the m^6^A site in the 3’-UTR of HK2 mRNA (near stop codon) (Fig. 5d). MeRIP-qPCR indicated that METTL3 knockdown decreased HK2 mRNA level precipitated by m^6^A antibody, while METTL3 overexpression promoted the level (Fig. 5e). Correlation analysis by Spearman’s rank correlation coefficient (GEPIA, http://gepia.cancer-pku.cn/) showed that METTL3 was positively correlated with HK2 expression in the CC tissue specimens (Fig. 5f). Overall, these findings support that HK2 acts as the target of METTL3 in CC cells.

### METTL3 recruits YTHDF1 to regulate HK2 mRNA stability

**Fig. 6:**
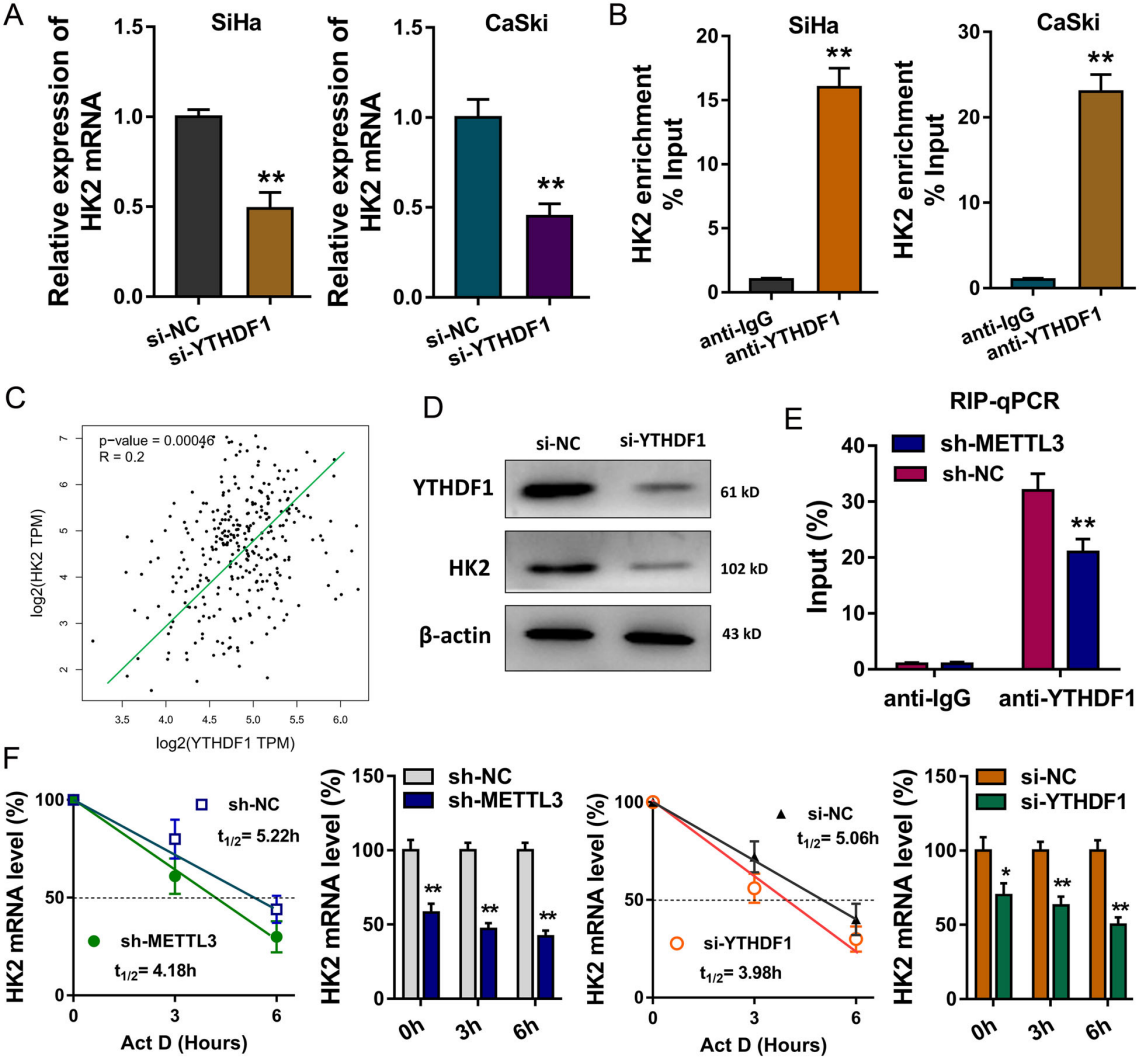
METTL3 recruits YTHDF1 to regulate HK2 mRNA stability. **a** RT-PCR demonstrated the HK2 mRNA expression in CC cells transfected with YTHDF1 silencing. **b** RNA immunoprecipitation (RIP)-PCR indicated the direct binding within YTHDF1 and HK2 mRNA. **c** Correlation analysis by Spearman’s rank correlation coefficient showed the positive correlation within HK2 expression and YTHDF1 in the CC tissue specimens. **d** Western blotting analysis indicated the protein production of HK2 with YTHDF1 silencing. **e** RIP-qPCR indicated the enrichment of HK2 mRNA in CC cells, using anti-YTHDF1 antibody, with METTL3 knockdown. **f** RNA decay rate followed by RT-PCR assay demonstrated the HK2 mRNA half-lives upon the METTL3 knockdown and YTHDF1 silencing. Data were detected at indicated timepoint with actinomycin D (Act D, 5 μg/ml) treatment. Data are displayed as mean ± standard deviation. **p < 0.01 vs. control. *p < 0.05 vs. control.

Existing finding indicated that HK2 acted as the target of METTL3; however, the deepgoing mechanism by which METTL3 promoted HK2 expression was still unclear. Previously researches inspired us that METTL3 could recruit YTHDF1 to enhance their target transcript stability. RT-PCR demonstrated that YTHDF1 silencing could reduce the HK2 mRNA expression, suggesting that YTHDF1 might participate in HK2 epigenetic regulation (Fig. 6a). RNA immunoprecipitation (RIP)-PCR indicated the direct binding within YTHDF1 and HK2 mRNA (Fig. 6b). Correlation analysis by Spearman’s rank correlation coefficient (GEPIA, http://gepia.cancer-pku.cn/) showed that YTHDF1 was positively correlated with HK2 expression in the CC tissue specimens (Fig. 6c). Western blotting analysis indicated that YTHDF1 silencing could repress the protein production of HK2 (Fig. 6d). Moreover, RNA immunoprecipitation followed by qPCR indicated that the direct interaction between YTHDF1 and HK2 mRNA was reduced by the METTL3 knockdown (Fig. 6e). RNA decay rate assay demonstrated that HK2 mRNA half-lives were significantly shortened upon the METTL3 knockdown and YTHDF1 silencing (Fig. 6f). Taken together, these findings confirmed that the methylated HK2 mRNA was recognized by YTHDF1, and METTL3/YTHDF1 enhanced its mRNA stability.

## Discussion

N^6^-methyladenosine (m^6^A) modification is a type of methylation on the 6th nitrogen position of adenosine base of mRNA^21^. The m^6^A modification is dynamically reversible on the mRNA, which was operated by methyltransferase or methyltransferase.

In human pathophysiology, METTL3 has been recognized as an essential factor, including diabetes, cancers and cardiovascular disease^22,23^. For example, in bladder cancer tumorigenesis, m^6^A methyltransferase METTL3 and demethylases ALKBH5 mediate the m^6^A modification in 3’-UTR of CDCP1 mRNA. METTL3 recruits m^6^A reader YTHDF1 to recognize m^6^A residues of CPCP1 3’-UTR, thereby promoting CDCP1 translation^24^. These finding might provide insight into critical roles of the METTL3/m^6^A/YTHDF1/CDCP1 axis in chemical carcinogenesis. Similarly, in bladder cancer, the METTL3-mediated m^6^A modification on the AFF4 could promote its expression. Besides, AFF4 binds to the promoter of MYC, suggesting the METTL3/m^6^A/AFF4/NF-κB/MYC axis for bladder cancer tumorigenesis^25^.

In present research, our data demonstrated that METTL3 was significantly upregulated in the CC tissue and cell lines. Besides, this ectopic overexpression was correlated with poor prognosis. Functional trials indicated that METTL3 could promote the proliferation and reduce the apoptosis of CC cells in vitro. Moreover, METTL3 accelerated the Warburg effect or aerobic glycolysis. These data indicated that METTL3 could act as the oncogenic element for the CC tumorigenesis. Moreover, the potential mechanism for the regulation mediate by METTL3 might the aerobic glycolysis pattern. In the existing research, the roles of METTL3 on the tumorous aerobic glycolysis are still infrequently reported. For example, METTL3 stimulates the m^6^A modification of HDGF mRNA, and the recruits m^6^A reader IGF2BP3 to directly recognize the m^6^A site and enhance HDGF mRNA stability. The nuclear HDGF could activate GLUT4 and ENO2, thereby increasing the glycolysis in CC cells^26^. What caught our attention was that our findings were accordance with this finding, supporting the critical roles of METTL3 in the cancerous Warburg effect. The m^6^A modification not only locates in the linear transcript (mRNA or noncoding RNA)^27,28^, but also locates in the circular transcript (circular RNA)^29^.

In this finding, we discovered that METTL3 positively enhanced the stability of HK2 mRNA to increase it protein expression, thereby accelerating the glycolysis process. Besides, we found that the interaction within METTL3 and HK2 was recognized and mediated by the m^6^A reader YTHDF1, which could recognize the m^6^A site in the HK2 mRNA and assist the binding of METTL3 towards HK2 mRNA. The interaction of METTL3/YTHDF1 complex and their roles on target transcripts have adequately identified. For example, in liver cancer, YTHDF1 mediates m^6^A-increased translation of Snail mRNA, and the methylation is catalyzed by METTL3, suggesting the acceleration mediated by METTL3/YTHDF1 complex for the methylated target mRNA^30^.

HK2 is one of the critical key enzymes for the Warburg effect or aerobic glycolysis. In the functional cellular experiments, we found that METTL3 knockdown or overexpression could remarkedly regulate the glycolysis elements, e.g., HK2, PKM2, and GLUT1. Moreover, in the MeRIP-Seq, we found that there was a remarkable m^6^A sites in the 3’-UTR of HK2 mRNA. MeRIP-qPCR and RIP-qPCR demonstrated that METTL3 recruited m^6^A reader YTHDF1 to install the methylation of HK2 mRNA to enhance its stability.

**Fig. 7:**
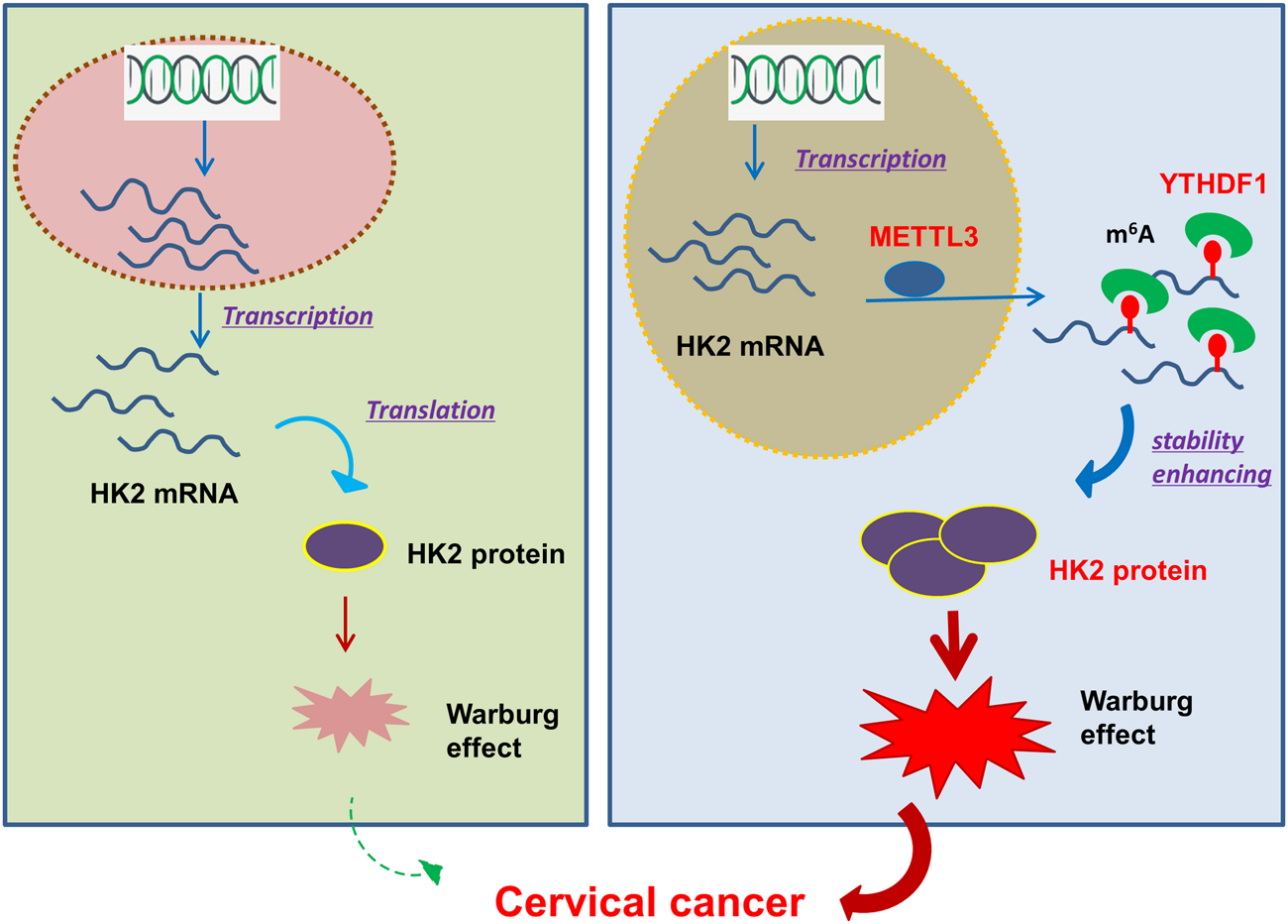
METTL3/YTHDF1/HK2 axis accelerated the CC carcinogenesis and Warburg effect.

In conclusion, these finding discovered the critical roles of METTL3 on the CC carcinogenesis and Warburg effect. Mechanistically, m^6^A-modified HK2 mRNA is regulated by an m^6^A reader protein YTHDF1, which recognizes m^6^A and enhances the stability of target transcripts. METTL3 recruits YTHDF1 to regulate HK2 mRNA stability, exerting an oncogenic factor via YTHDF1/HK2 axis by accelerating glycolysis, which providing a potential prognostic biomarker for CC (Fig. 7).

## Supplementary information

Table S1
