# Supplementary material for: N6-methyladenosine METTL3 promotes cervical cancer tumorigenesis and Warburg effect through YTHDF1/HK2 modification
Source: Cell Death Dis. 2020 Oct 24;11(10):911. doi: 10.1038/s41419-020-03071-y (PMC7585578; doi:10.1038/s41419-020-03071-y)
Supplement: Supplementary file 1 — Table S1 [file 41419_2020_3071_MOESM1_ESM.docx]

**Supplement Table 1**. Sequences of shRNA and qRT-PCR primers.

|  | 5’-3’ |
| --- | --- |
| METTL3 | forward, 5’-TTGTCTCCAACCTTCCGTAGT-3'  reverse, 5'-CCAGATCAGAGAGGTGGTGTAG-3’ |
| YTHDF1 | forward, 5’-ACCTGTCCAGCTATTACCCG-3'  reverse, 5'-TGGTGAGGTATGGAATCGGAG-3' |
| HK2 | forward, 5’-GAGCCACCACTCACCCTACT-3'  reverse, 5'-CCAGGCATTCGGCAATGTG-3' |
| sh-METTL3-1 | 5’- CCGGGCCTTAACATTGCCCACTGATCT CGAGATCAGTGGGCAATGTTAAGGCTTTTTG-3’ |
| sh-METTL3-2 | 5’- CCGGGCAAGTATGTTCACTATGAAACTCGAG TTTCATAGTGAACATACTTGCTTTTTG-3’ |
| sh-METTL3-3 | 5’- CCGGGCTGCACTTCAGACGAATTATCTCGA GATAATTCGTCTGAAGTGCAGCTTTTTG-3’ |
| beta-actin | forward, 5’-CTCCATCCTGGCCTCGCTGT-3’  reverse, 5’-GCTGTCACCTTCACCGTTCC-3’ |
